# The impact of red meat and processed meat consumption on the risk of development and relapse of ulcerative colitis: a systematic review and dose-response meta-analysis

**DOI:** 10.3389/fnut.2025.1668302

**Published:** 2025-09-12

**Authors:** Yangyang Zhang, Yitong Yu, Ziyun Jiang, Junhong Yu, Zeyang Zhang, Zhuojia An, Yanhong Du, Yiqing Mao, Lanshuo Hu, Xudong Tang, Yingpan Zhao, Tangyou Mao

**Affiliations:** ^1^Institute of Digestive Diseases, Xiyuan Hospital, China Academy of Chinese Medical Sciences, Beijing, China; ^2^Graduate School of China Academy of Chinese Medical Sciences, Beijing, China; ^3^Dongfang Hospital, Beijing University of Chinese Medicine, Beijing, China; ^4^Department of Epidemiology and Biostatistics, School of Public Health, Peking University Health Science Center, Beijing, China; ^5^Centre for Evidence-Based Chinese Medicine, Beijing University of Chinese Medicine, Beijing, China; ^6^Graduate College, Beijing University of Chinese Medicine, Beijing, China; ^7^Beijing Hospital of Traditional Chinese Medicine, Beijing, China

**Keywords:** red meat, processed meat, ulcerative colitis, systematic review, dose-response meta-analysis

## Abstract

**Background:**

Consumption of red and processed meats has been classified as probably carcinogenic and carcinogenic to humans, respectively. However, the association between their consumption and the incidence or recurrence of ulcerative colitis (UC) remains unclear. This study aims to systematically evaluate the dose–response relationship between red or processed meat consumption and UC.

**Methods:**

Databases including PubMed, Cochrane Library, Web of Science, Embase, CNKI, VIP, Wanfang, SinoMed, Yiigle, and ICTRP were searched from inception through July 2024. Pooled relative risks (RRs) with 95% confidence intervals (CIs) were estimated using random-effects or fixed-effects models based on heterogeneity. A dose-response meta-analysis was conducted using R 4.4.2.

**Results:**

Eighteen studies comprising 1,384,024 participants were included, all rated as moderate to high quality. Red meat consumption was significantly associated with an increased risk of UC development [RR = 1.21, 95% CI (1.03, 1.42), *p* = 0.020]. Processed meat consumption showed a tendency toward increased UC risk, although not statistically significant [RR = 1.54, 95% CI (0.99, 2.42), *p* = 0.058]. Neither red nor processed meat consumption was significantly associated with UC recurrence. Dose–response analysis indicated that each additional 100 g/day of red meat intake increased UC incidence risk by 65% [RR = 1.65, 95% CI (1.30, 2.09)].

**Conclusion:**

Based on very low-certainty evidence, increased red meat intake may be associated with a potential risk of developing UC. However, there is currently insufficient evidence to support an association between red or processed meat consumption and the recurrence of UC. Future studies with long-term follow-up and rigorous design are warranted to verify these findings and explore underlying mechanisms.

**Systematic Review Registration:**

https://www.crd.york.ac.uk/PROSPERO/view/CRD42024573557, identifier (CRD42024573557).

## 1 Introduction

Ulcerative colitis (UC) is a chronic inflammatory disease characterized by continuous and diffuse inflammation predominantly affecting the mucosal and submucosal layers of the colon and rectum (1). Clinically, UC commonly presents as bloody diarrhea with alternating periods of relapse and remission (2). UC significantly impairs patients’ quality of life and may progress to colorectal cancer or mortality in severe cases (3). Epidemiological studies indicate a particularly high prevalence of UC in Western countries, with reported rates ranging from 286 to 500 cases per 100,000 individuals in Europe (4). In recent years, a notable rise in UC incidence has been observed in Asian countries, further exacerbating the global burden of the disease (5). Although dietary habits, environmental exposures, and genetic susceptibility are implicated in the etiology of UC, the exact pathogenesis remains poorly understood (6). UC typically follows a recurrent and progressive clinical course characterized by repeated exacerbations (7). Some studies have suggested potential associations between specific dietary factors or medications and UC relapses; however, these associations remain to be fully clarified (8).

According to the NOVA classification system, red meat refers to unprocessed or simply cooked mammalian muscle meat, such as beef, lamb, and pork. Processed meat refers to meat products that have been salted, cured, fermented, smoked, or treated with food additives such as emulsifiers, sweeteners, and colorants—examples include bacon, sausages, and canned meat (9). The International Agency for Research on Cancer classifies red meat as a Group 2A carcinogen and processed meat as a Group 1 carcinogen (10). Dietary guidelines regarding red and processed meat consumption vary significantly among countries and regions. Approximately 23% of countries, primarily in Europe, provide qualitative or quantitative guidance recommending reduced consumption of red and processed meat. In contrast, most Asia-Pacific countries have not specifically recommended limiting red meat intake (11). Similarly, nutritional guidelines for UC patients also show inconsistency. The International Organization for the Study of Inflammatory Bowel Disease recommends reducing red and processed meat consumption for UC patients, based on very low certainty evidence (12). Nevertheless, other studies suggest that restricting red meat intake may negatively impact patients’ health and conflict with their dietary preferences (13). As a result, clinicians often encounter challenges when providing specific nutritional recommendations.

We hypothesize that the influence of meat consumption on the incidence and recurrence of UC varies depending on the type and quantity of meat consumed. An existing systematic review suggests that each 100 g increase in meat intake raises the risk of inflammatory bowel disease by 38% (14). Nevertheless, there is currently a lack of systematic reviews specifically examining the relationship between red or processed meat intake and UC incidence or recurrence. Therefore, this study aims to systematically review the association between red and processed meat consumption and the risks of UC incidence and recurrence, analyzing the dose–response relationship to provide evidence-based dietary recommendations for UC patients.

## 2 Methods

This systematic review was conducted following the Preferred Reporting Items for Systematic Reviews and Meta-Analyses (PRISMA) 2020 guidelines and was prospectively registered on PROSPERO (registration number: CRD42024573557).

### 2.1 Search strategy

A comprehensive literature search was conducted in PubMed, Embase, Cochrane Library, Web of Science, China National Knowledge Infrastructure (CNKI), Chongqing VIP Chinese Science and Technology Journal Database, Wanfang Database, Chinese Biomedical Literature Database (CBM), and Yiigle Database from their inception until July 22, 2024, limited to studies published in Chinese or English. The search strategy utilized terms including “red meat,” “beef,” “mutton,” “colitis, ulcerative,” and “ulcerative colitis.” Detailed search strategies are provided in Supplementary Table S1. To minimize the risk of missing relevant studies, additional manual screening of reference lists from included studies and pertinent reviews was performed, complemented by expert consultation.

### 2.2 Inclusion and exclusion criteria

The inclusion criteria were established according to the Population, Exposure, Comparator, Outcomes, and Study Design (PECOS) framework. Detailed inclusion criteria are presented in Table 1. The following studies were excluded: (1) studies evaluating dietary patterns explicitly controlling for red or processed meat intake, such as the Mediterranean diet or low-fat diet; (2) studies without clear specification of nutrient origin; and (3) duplicate publications or studies where the full text was inaccessible.

**Table 1:** PECOS criteria for inclusion of studies.

Parameter	Criterion
Population	Ulcerative colitis patients diagnosed by specific criteria
Exposure	Varying levels of red meat or processed meat consumption
Comparison	Comparison of levels/different amounts of red meat or processed meat consumption
Outcomes	The occurrence risk of ulcerative colitis The recurrence risk of ulcerative colitis
Study design	Cohort study, case–control study

### 2.3 Literature screening and data extraction

Retrieved literature was imported into NoteExpress 3.4 software, and duplicate publications were removed. Two reviewers (YYZ, ZYJ) independently conducted initial screening based on titles and abstracts, followed by a comprehensive full-text review. Subsequently, reviewers (ZJA, YHD, ZYZ, ZYJ) independently extracted data using a pre-designed data extraction form. The data extraction form included study identification, study design, sample size, participant demographics (age, sex), diagnostic criteria, country, type and dose of red or processed meat, dietary assessment tools, follow-up duration, effect sizes [odds ratio (OR), hazard ratio (HR), or relative risk (RR)] with their 95% confidence intervals (CIs), and adjustment factors. When multiple estimates with different adjustments were available, the estimate adjusted for the most covariates was selected. Any discrepancies during screening or data extraction were resolved by consulting a third reviewer (TYM).

### 2.4 Quality assessment

The quality of the included studies was assessed independently by two reviewers (YYZ, JHY) using the Newcastle-Ottawa Scale (NOS) for cohort and case–control studies (15). The NOS evaluates three domains: selection of study population, comparability of groups, and assessment of exposure or outcomes. A maximum of 9 points can be awarded using the NOS, with scores ≥7 considered high quality, 5–6 as moderate quality, and <5 as low quality (16). Two reviewers (YYZ, JHY) also independently assessed the certainty of evidence for the main findings using the Grading of Recommendations, Assessment, Development, and Evaluation (GRADE) approach (17). The certainty of evidence was evaluated across five domains for downgrading (risk of bias, inconsistency, indirectness, imprecision, and publication bias) and three domains for upgrading (large magnitude of effect, dose–response gradient, and effect of plausible confounding). Any disagreements were resolved through discussion with a third reviewer (TYM).

### 2.5 Data synthesis and analysis

All statistical analyses were conducted using the “meta” and “metafor” packages in R version 4.4.2 within the R Studio environment. For the meta-analysis, the highest category of red or processed meat intake was compared to the lowest (reference group) using a random-effects model. Given the low incidence of UC (below 10%), the HR and OR are considered to approximate the RR. Therefore, both HR and OR were treated as equivalent to RR for the purpose of effect size pooling in this meta-analysis (18). Heterogeneity was assessed using the Q test and the *I^2^* statistic, with *I^2^* < 30% indicating low heterogeneity. A two-sided *p*-value < 0.05 was considered statistically significant.

Where data from studies with more than three intake categories were available, a dose-response meta-analysis was conducted. Generalized least squares regression and restricted cubic spline models with knots at the 10th, 50th, and 90th percentiles were applied. Intake levels were estimated based on reported upper and lower category boundaries. For open-ended intake ranges, it was assumed that the width of the interval equaled that of the adjacent category. When portion sizes were reported instead of weight, one serving was standardized to 100 g of red meat and 50 g of processed meat (19).

Subgroup analyses and meta-regression were performed to explore sources of heterogeneity, considering variables such as study design, dietary pattern (Western vs. Eastern), meat type, dietary assessment method, outcome definition, and adjustment for confounding variables. Where applicable, sensitivity analyses were conducted to assess the robustness of the findings. For meta-analyses including more than 10 estimates, publication bias was assessed using funnel plots and Egger’s regression test.

## 3 Results

### 3.1 Literature screening and characteristics of included studies

**Figure 1:**
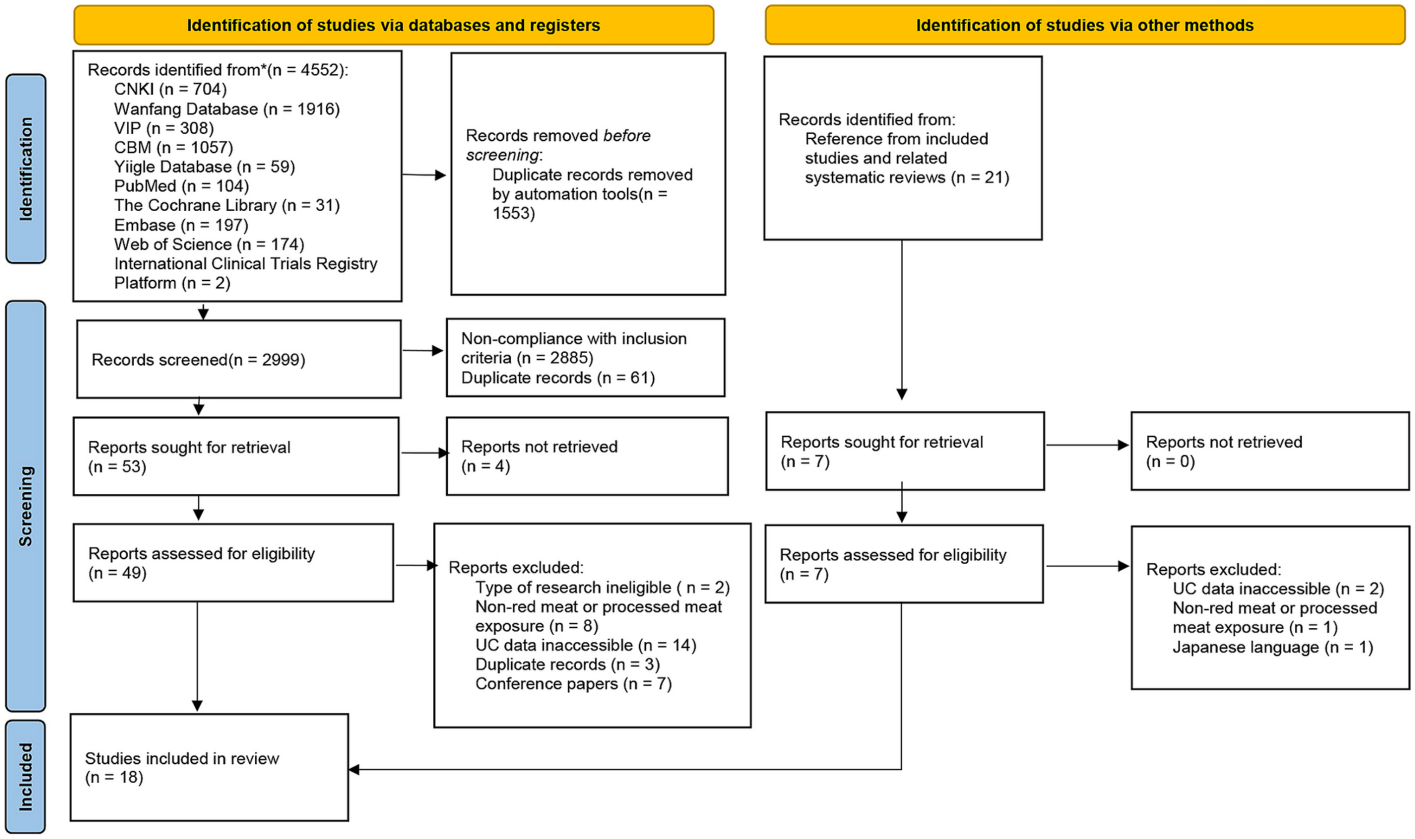
Flow diagram of study selection in this review. CNKI, the Chinese National Knowledge Infrastructure Databases; VIP, the Chongqing Chinese Science and Technology Journal Database; CBM, Chinese Biomedical Literature Database; n, number; UC, ulcerative colitis.

A total of 4,552 records were identified through database searches, and an additional 21 records were retrieved via manual searches. After automated deduplication, 2,999 unique records remained. Following title and abstract screening, 53 full-text articles were retrieved. After excluding ineligible studies (Supplementary Table S2), 18 studies were included in the final analysis, including 13 investigating UC incidence and 5 addressing UC recurrence (Figure 1).

The final analysis comprised 18 studies published between 1993 and 2024, including 9 cohort studies and 9 case–control studies, with a combined sample size of 1,384,024 participants. Seventeen studies were published in English and one in Chinese. Eleven studies focused on populations from Western countries characterized by Western dietary patterns, while seven included participants from Asian countries with Eastern dietary habits. Ten studies examined processed meat consumption, fifteen investigated red meat intake, and one specifically evaluated pork consumption during childhood (Tables 2, 3). These studies accounted for a range of confounders, including geographic region, age, sex, smoking status, energy intake, and physical activity, as detailed in Supplementary Tables S3 and S4.

**Table 2:** Characteristics of included studies for the risk of development of ulcerative colitis.

Study	Total sample size	Sample size (UC)	Age (mean ± SD, years)	Sex (M/F)	Country	Cohort	Exposure	Dietary assessment tool	Outcome measurement	Lowest vs. highest intake assessment	Follow-up period (mean, years)	Person-years
Cohort studies												
Dong et al. (26)	413,593	418	52.5 ± 8.6	128,214/285379	Eight countries^a^	EPIC-IBD	Meat/red meat/processed meat	①	B	M: 0-19 g/d vs.>61 g/d; F: 0-10 g/d vs.>38 g/d	16.8	6,961,118.6
Lopes et al. (27)	208,070	456	45.31 ± 10.70	41,871/166199	America	NHS, NHSII, HPFS	Red meat	②	A	NR	NR	5,117,021
Narula et al. (28)	116,037	377	50.2 ± 9.7	47,305/68732	21 countries^b^	PURE	Processed meat	①	A	<1 serving/week vs. ≥7 servings/week	Median: 9.7	NR
DeClercq et al. (29)	12,568	120	UC: 54.6 ± 8.1 C: 53.7 ± 8.8	UC: 42/77 C:3694/8768	Canada	PATH	Meat and poultry	③	A	Rarely/never vs. servings per month	NR	NR
Khalili et al. (30)	165,331	321	42.4	0/165331	USA	NHS, NHSII	Red meat/processed meat	②	A	NR	NR	3,038,049
Song et al. (31)	456,590	312	Total: 51.8 UC: 61.3	258,887/197703	China	CKB	Meat	②	A	Never, rarely or monthly vs. ≥4 days per week	12.1	Never, rarely or monthly: 924,568 1–3 days per week: 1,896,199 ≥ 4 days per week: 2,535,737
Case–control studies												
Bernstein et al. (32)	1,014	217	Range: 18–50	UC: 99/118 C: 115/318	Canada	NA	Eating pork as a child	④	A	Never or less than once a month vs. 3–6 times a week or every day	NA	NA
Farsiz et al. (33)	234	86	UC: 40.8 ± 12.7 C: 36.4 ± 11.6	UC: 32/54 C: 49/73	Iran	NA	Red meat/processed meat	③	A	NR	NA	NA
Kurata (34)	244	101	Range: 10–39	UC: 56/45 C: 79/64	Japan	NA	Meat/ham and sausage	⑤	B	None or hardly any vs. three to five times per week	NA	NA
Liu (35)	216	72	Median: UC: 43/T:45	UC: 32/40 C: 64/80	China	NA	Red meat/grilled meat	②	A	0–3 serving/week vs. ≥5 serving/week	NA	NA
Sakamoto et al. (36)	445	108	Range: 15–34	UC: 56/52 C: 135/76	Japan	NA	Meats and poultry	①	A	0 g/d vs. 94.6 g/d	NA	NA
Rashvand et al. (37)	186	62	UC: 37.4 C: 36.23	UC: 27/35 C: 54/70	Iran	NA	Red meat/processed meat	①	A	NR	NA	NA
Maconi et al. (38)	243	41	Total: 37.5 ± 15.2 C: 40.4 ± 14.6	Total: 49/34 C: 97/63	Italy	NA	Red meat	①	B	NR	NA	NA

UC, ulcerative colitis; SD, standard deviation; M, male; F, female; C, control group; NR, not reported; NA, not applicable; vs., versus; g, gram; d, day; NHS, the Nurses’ Health Study; HPFS, Health Professionals Follow-Up Study; EPIC, the European Prospective Investigation into Cancer and Nutrition; PURE, the Prospective Urban Rural Epidemiology; PATH, the Atlantic Partnership for Tomorrow’s Health; CKB, the China Kadoorie Biobank. ①, country-specific validated food frequency questionnaires; ②, semi-quantitative food frequency questionnaires; ③, food frequency questionnaires; ④, questionnaire about childhood dietary patterns; ⑤, self-administered questionnaire; A, self-reported questionnaire; B, individual interviews or self-reported questionnaire.

a Denmark, France, Germany, Italy, The Netherlands, Spain, Sweden, and the UK.

b Argentina, Bangladesh, Brazil, Canada, Chile, China, Colombia, India, Iran, Malaysia, Palestine, Pakistan, Philippines, Poland, South Africa, Saudi Arabia, Sweden, Tanzania, Turkey, United Arab Emirates, and Zimbabwe.

**Table 3:** Characteristics of included studies for the risk of flare of ulcerative colitis.

Study	Total sample size	Sample size (flare/UC)	Age (mean ± SD, years)	Sex (M/F)	Country	Cohort	Exposure	Dietary assessment tool	Outcome measurement	Flare assessment tool	Definition of flare	Lowest vs. highest intake assessment	Follow-up period (mean, years)
Cohort studies													
Cohen et al. (39)	6,768	185/597	44.9	214/383	USA	CCFA	Red meat/processed meat	②	A	5-point Likert scale	Patients with active disease activity were those who reported having mild, moderate, or severe symptoms.	NR	NA
Barnes et al. (40)	412	45/412	Flare: 44.61 ± 12.64 Remission: 48.66 ± 14.71	Flare: 28/17 Not Remission: 207/158	USA	A consortium of academic and community gastroenterology practices	Processed meat	①	A	SCCAI	A SCCAI ≥5 or a change in disease activity requiring a change in medication.	Never or less than once per month vs. 6 or more times per day	1
Jowett et al. (41)	183	96/183	Median:51	93/90	UK	NR	Meat and meat products/red and processed meat	③	A	SCCAI	A score of 5 or more accurately confirms a clinician defined relapse	NR	1
Case–control studies													
Mi et al. (42)	100	27/40	Flare: 53.2 ± 12.2 Remission: 48.4 ± 15.8	Flare: 20/11 Remission: 11/8	China	NR	Beef	①	A	Modified Truelove and Witts severity index	UC relapse refers to the recurrence of UC symptoms following natural or drug treatment into the remission period.	Not at all or occasionally vs. ≥3times per week	NA
Peters et al. (43)	1790	94/207	Flare: 43.2 ± 15.1 Remission: 42.1 ± 13.7	Flare: 92/149 Remission:100/151	Dutch	1000IBD, LLD	Meat	②	A	SCCAI	Relapse was defined as either a faecal calprotectin level≥200 mg/g or a SCCAI>2.	NR	NA

UC, ulcerative colitis; SD, standard deviation; M, male; F, female; NR, not reported; NA, not applicable; vs., versus; g, gram; d, day; CCFA, the Crohn’s and Colitis Foundation of America; SCCAI, Short Clinical Colitis Activity Index; 1000IBD, the Groningen 1000IBD cohort; LLD, the Lifelines DEEP Cohort; ①, country-specific validated food frequency questionnaires; ②, semi-quantitative food frequency questionnaires; ③, food frequency questionnaires; ④, questionnaire about childhood dietary patterns; ⑤, self-administered questionnaire; A, self-reported questionnaire; B, individual interviews or self-reported questionnaire.

### 3.2 Risk of bias assessment

**Figure 2:**
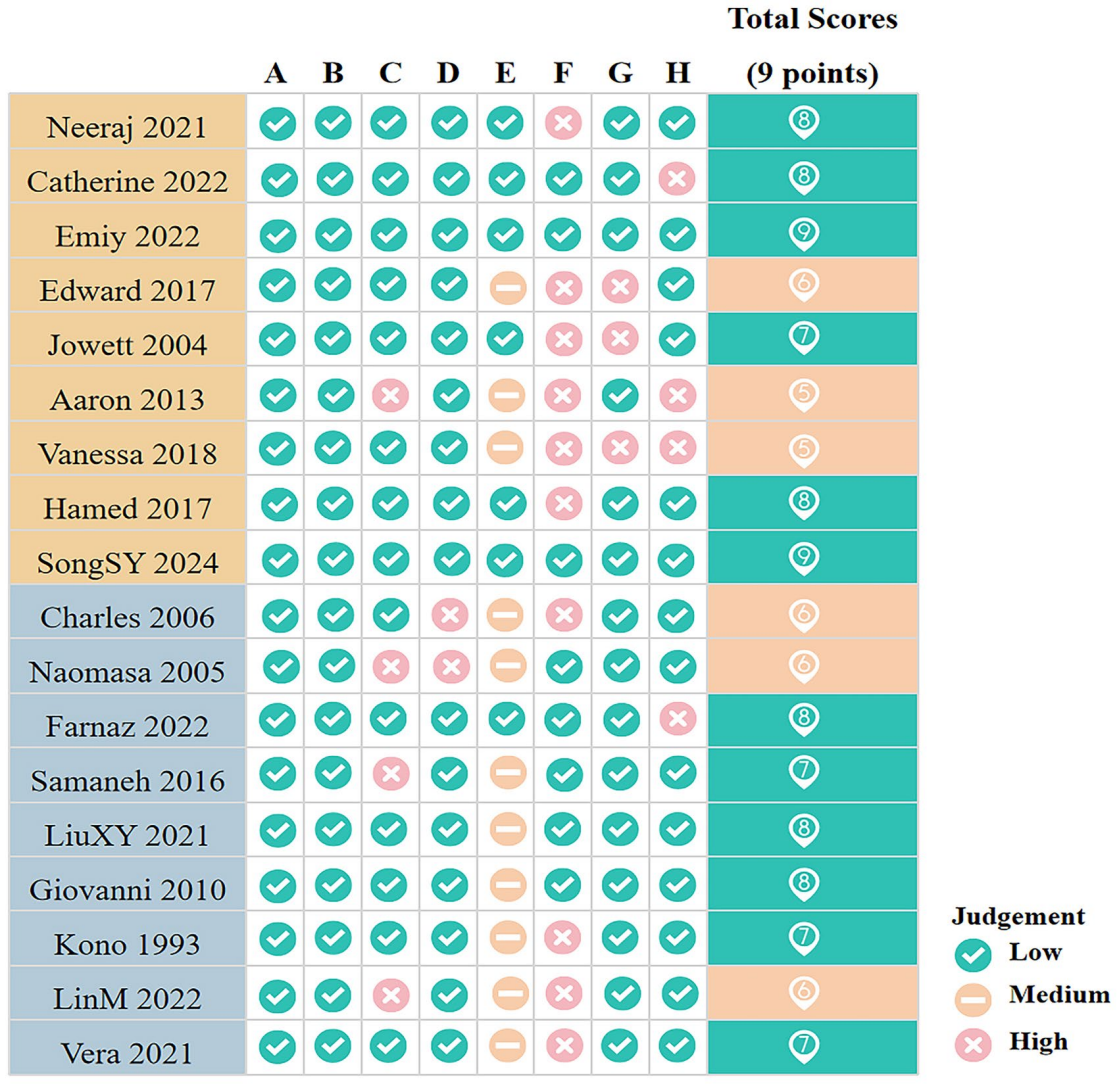
Quality assessment results for included studies. A, Representativeness of the exposed cohort/Is the case definition adequate (1 point); B, Selection of the non exposed cohort/Representativeness of the cases (1 point); C, Ascertainment of exposure/Selection of Controls (1 point); D, Demonstration that outcome of interest was not present at start of study (1 point)/Definition of Controls; E, Comparability of cohorts on the basis of the design or analysis/Comparability of cases and controls on the basis of the design or analysis (2 points); F, Assessment of outcome/Ascertainment of exposure (1 point); G, Was follow-up long enough for outcomes to occur/Same method of ascertainment for cases and controls (1 point); H, Adequacy of follow up of cohorts/Non-Response rate (1 point). The green background indicates high-quality studies; the orange background indicates moderate-quality studies.

The results of the quality assessment are presented in Figure 2. Among both cohort and case–control studies, three were rated as moderate quality and six as high quality. The mean quality score was 7.22 for cohort studies and 7.00 for case–control studies. Overall, the included studies were predominantly of high methodological quality.

### 3.3 Meta-analysis of red meat consumption and the development risk of UC

**Figure 3:**
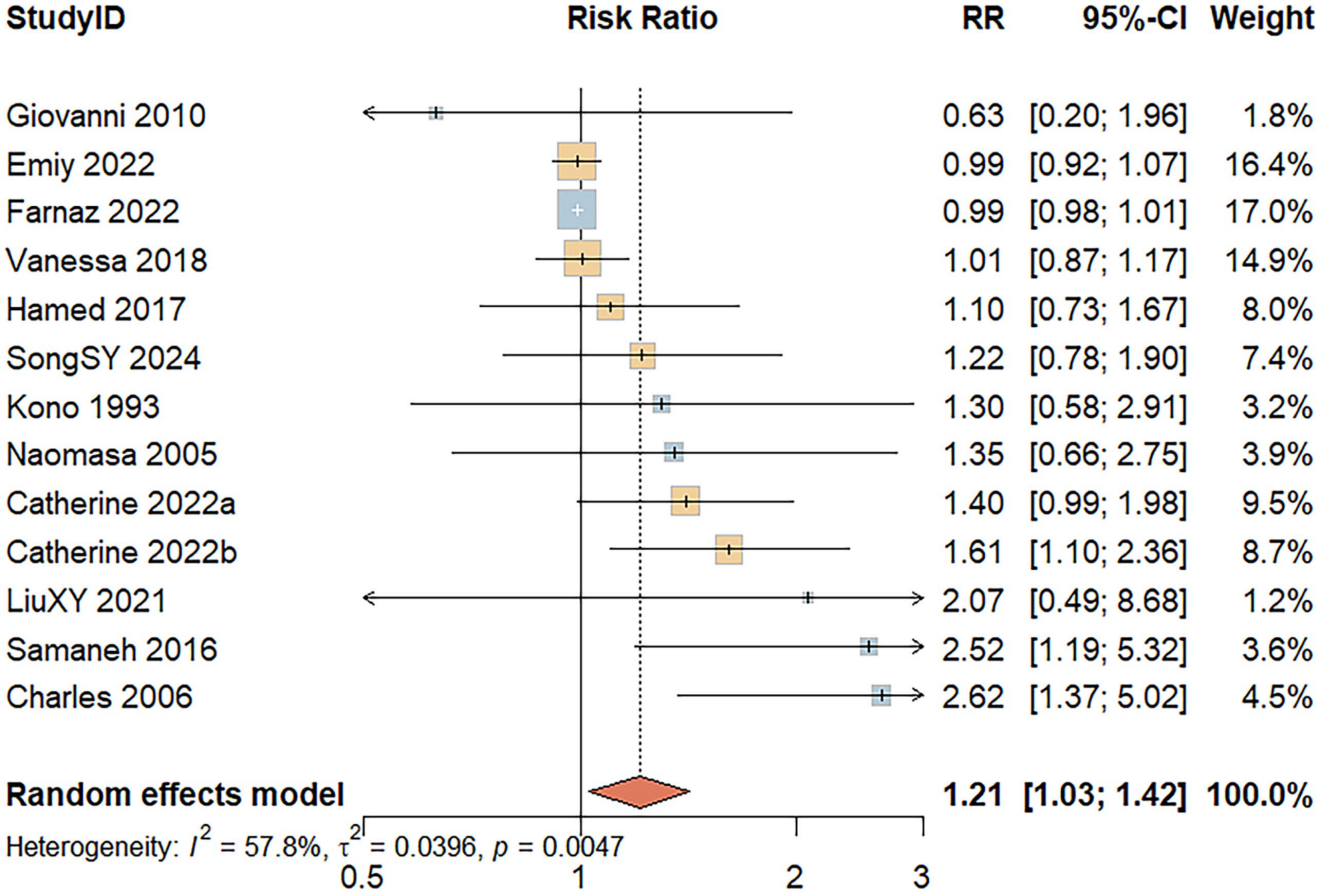
Forest Plot of the relationship between red meat consumption and the risk of development of UC.

Thirteen studies reported the association between red meat consumption and the risk of developing UC. A random-effects model revealed significant heterogeneity among the included studies (*I^2^* = 57.8%, *p* = 0.005). As shown in Figure 3, individuals with high red meat intake had a significantly increased risk of developing UC compared to those with low intake (RR = 1.21; 95% CI: 1.03–1.42; *p* = 0.020).

### 3.4 Meta-analysis of processed meat consumption and the development risk of UC

**Figure 4:**
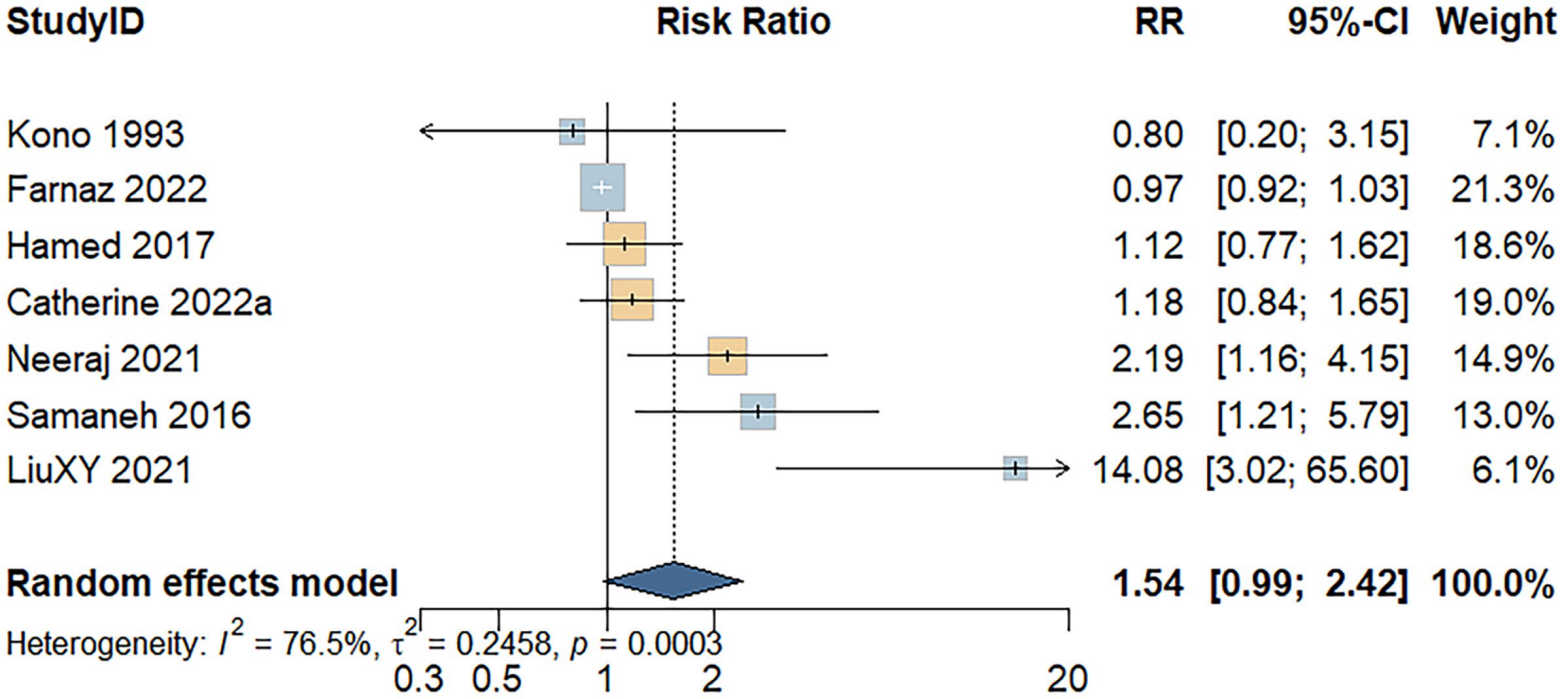
Forest plot of the relationship between processed meat consumption and the risk of development of UC.

Seven studies assessed the association between processed meat consumption and the risk of developing UC. A random-effects model indicated substantial heterogeneity among the studies (*I^2^* = 76.5%, *p* < 0.001). As shown in Figure 4, individuals with high processed meat consumption exhibited a non-significant increase in UC risk compared to those with low consumption (RR = 1.54; 95% CI: 0.99–2.42; *p* = 0.058).

### 3.5 Meta-analysis of red meat consumption and the recurrence risk of UC

**Figure 5:**
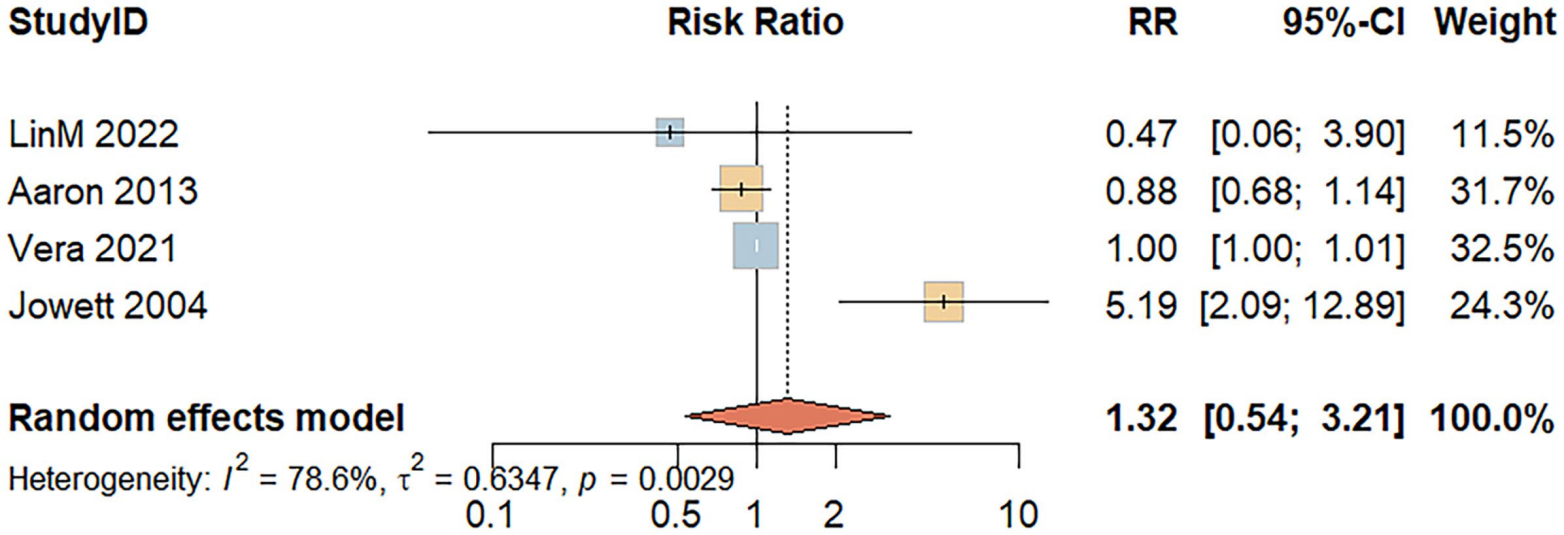
Forest plot of the relationship between red meat consumption and the risk of relapse of UC.

Four studies evaluated the association between red meat consumption and the risk of UC recurrence. A random-effects model indicated substantial heterogeneity among the included studies (*I^2^* = 78.6%, *p* = 0.003). As shown in Figure 5, the analysis revealed no significant association between red meat intake and UC recurrence risk (RR = 1.32; 95% CI: 0.54–3.21; *p* = 0.546).

### 3.6 Meta-analysis of processed meat consumption and the recurrence risk of UC

**Figure 6:**
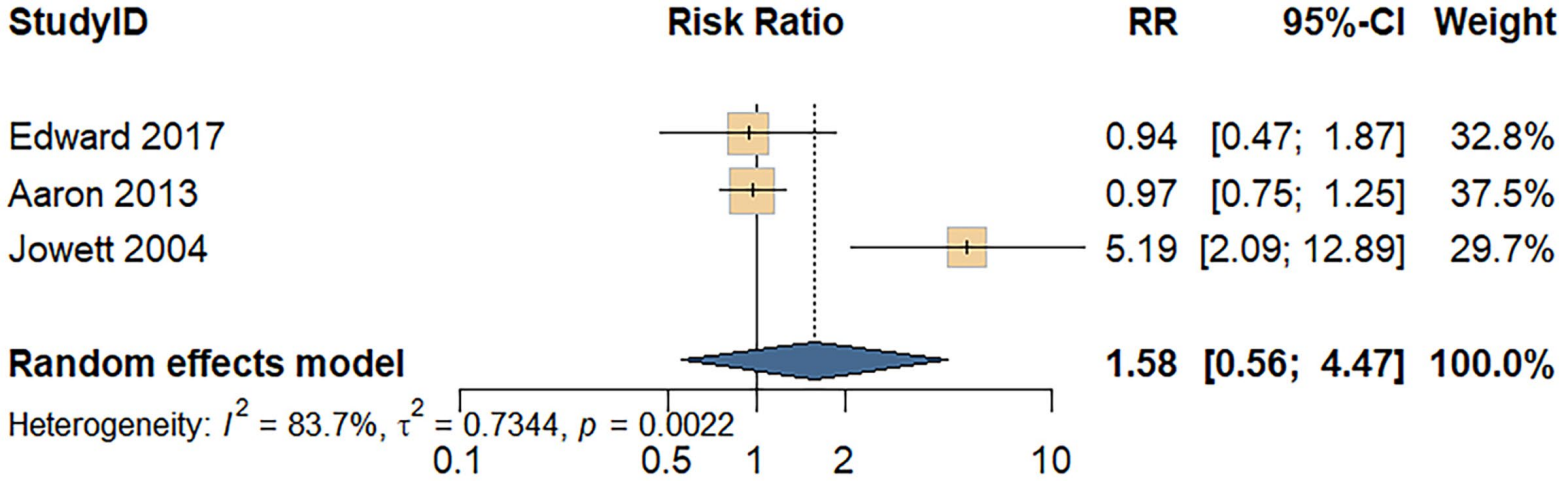
Forest plot of the relationship between processed meat consumption and the risk of relapse of UC.

Three cohort studies assessed the association between processed meat consumption and the risk of UC recurrence. A random-effects model revealed considerable heterogeneity among the studies (*I^2^* = 83.7%, *p* = 0.002). As shown in Figure 6, the meta-analysis revealed no significant difference in UC recurrence risk between individuals with high versus low processed meat intake (RR = 1.58; 95% CI: 0.56–4.47; *p* = 0.390).

### 3.7 Dose-response meta-analysis of red meat consumption and the development risk of UC

**Figure 7:**
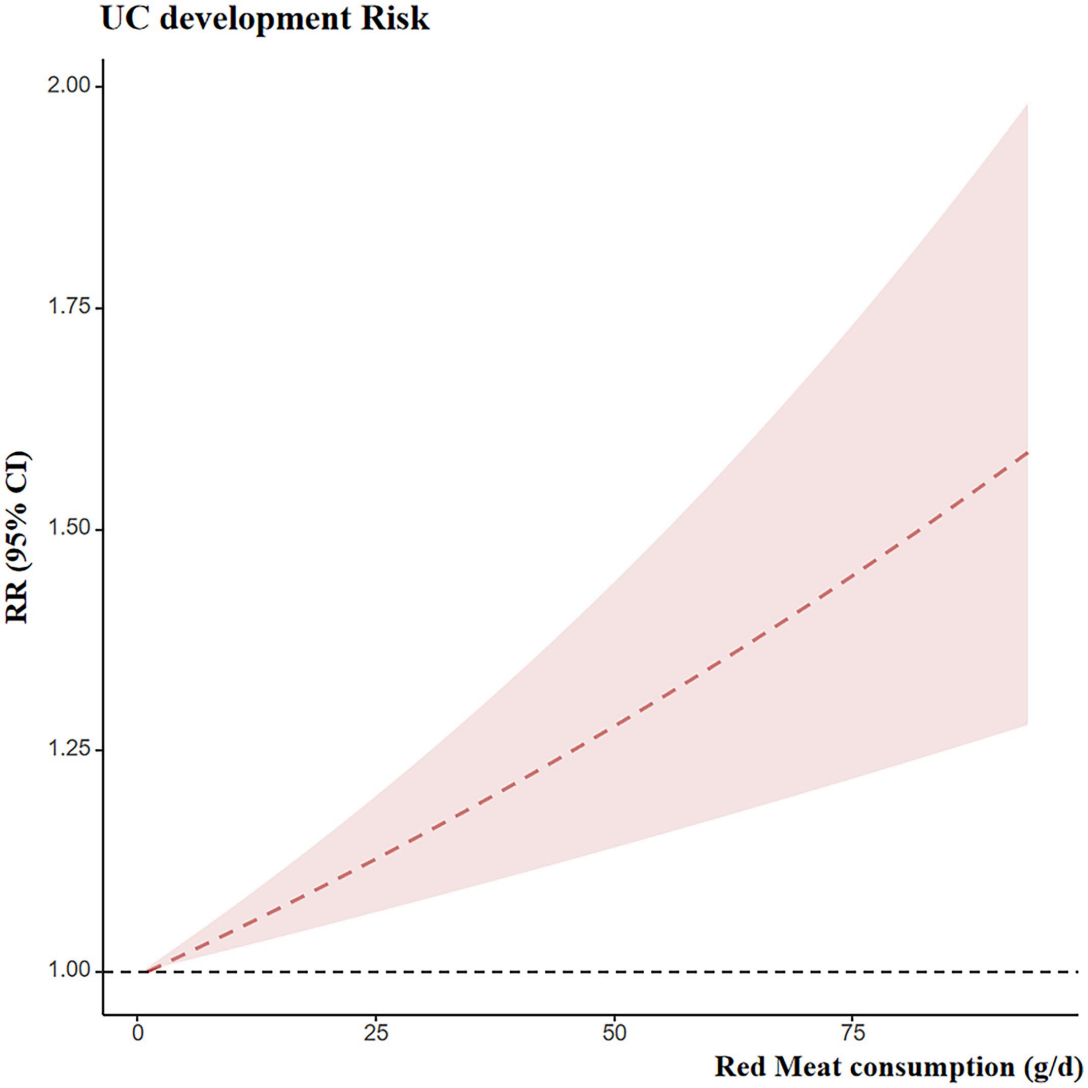
Dose–response curves for UC development risk and red meat exposure. RR, risk ratio.

Eight studies provided dose–response data suitable for analysis. The preceding meta-analysis suggested that red meat consumption may increase the risk of developing UC. Therefore, a dose–response analysis was conducted. A linear association was observed between red meat intake and UC risk, with a positive dose–response trend, as shown in Figure 7. Specifically, each 100 g/day increase in red meat intake was associated with an approximately 65% higher risk of UC (RR = 1.65; 95% CI: 1.30–2.09).

### 3.8 Subgroup analysis and meta-regression

Considerable heterogeneity was observed among studies investigating the association between red and processed meat consumption and the risk of UC. To explore potential sources of this heterogeneity, subgroup analyses were conducted based on study design, dietary characteristics, meat type, dietary assessment tools, outcome measurements, and adjusted confounders. These variables were also included as covariates in a meta-regression analysis.

Table 4 and Supplementary Figure S1 present the results of the subgroup analysis for the association between red meat consumption and UC risk. The findings suggest that heterogeneity may be partially explained by the type of dietary assessment tool used. In studies using country-specific validated food frequency questionnaires, no significant heterogeneity was observed, and red meat intake was significantly associated with increased UC risk (RR = 1.501; 95% CI: 1.160–1.941; *p* = 0.002). Outcome measurement, geographical region, and family history of inflammatory bowel disease may also contribute to the observed heterogeneity. However, meta-regression analysis did not identify any variables that significantly explained the heterogeneity.

**Table 4:** Subgroup analysis and meta-regression of the association between red meat consumption and the risk of development of ulcerative colitis.

Subgroup	*N*	Heterogeneity test		Estimate		Meta regression
		*I^2^*	*p*	RR (95%CI)	*p*	
Study design						0.457
Cohort study	6	89.00%	<0.001	1.242 (1.026, 1.504)	0.026	
Case control study	7	99.90%	<0.001	1.712 (1.220, 2.403)	0.002	
Dietary characteristics						0.845
Western-style diet	7	89.20%	<0.001	1.256 (1.071, 1.473)	0.005	
Eastern-style diet	6	99.30%	<0.001	1.221 (0.783, 1.903)	0.379	
Meat type						0.377
Meat	5	59.90%	0.041	1.268 (0.922, 1.744)	0.144	
Red meat	7	99%	<0.001	1.213 (1.012, 1.455)	0.037	
Eating pork as a child	1	/	/	2.620 (1.367, 5.020)	0.004	
Dietary assessment tool						0.623
T1	5	10.30%	0.347	1.501 (1.160, 1.941)	0.002	
T2	4	77.40%	0.004	1.134 (0.875, 1.470)	0.34	
T3	2	78.80%	0.03	1.007 (0.872, 1.162)	0.927	
T4	1	/	/	2.620 (1.367, 5.020)	0.004	
T5	1	/	/	1.300 (0.581, 2.907)	0.025	
Outcome measurement						0.052
A	9	97.60%	<0.001	1.500 (1.160, 1.941)	0.002	
B	4	0.00%	0.449	1.134 (0.879, 1.462)	0.334	
Adjusted factors						
Geographical region						0.945
Yes	3	0%	0.787	1.101 (0.728, 1.664)	0.648	
No	10	99.20%	<0.001	1.266 (1.026, 1.563)	0.028	
Family history of IBD						0.562
Yes	2	1.10%	0.315	0.991 (0.919, 1.068)	0.809	
No	11	99.20%	<0.001	1.300 (1.035, 1.634)	0.024	
Physical activity						0.034
Yes	6	99.50%	<0.001	1.255 (1.070, 1.473)	0.005	
No	7	44.40%	0.095	1.222 (0.785, 1.904)	0.374	

N, number; IBD, inflammatory bowel disease; RR, inrelative risk; T1, country-specific validated food frequency questionnaires; T2, semi-quantitative food frequency questionnaires; T3, food frequency questionnaires; T4, questionnaire about childhood dietary patterns; T5, self-administered questionnaire; A, self-reported questionnaire; B, individual interviews or self-reported questionnaire.

Table 5 and Supplementary Figure S2 summarize the subgroup analysis results for the association between processed meat consumption and UC risk. Heterogeneity in these studies may be influenced by study design, dietary characteristics, and the type of dietary assessment tool. In studies focusing on Western-style diets, processed meat consumption was significantly associated with increased UC risk (RR = 1.292; 95% CI: 1.012–1.649; *p* = 0.040). Similarly, in studies using country-specific validated food frequency questionnaires, processed meat intake significantly increased UC risk (RR = 1.352; 95% CI: 1.003–1.821; *p* = 0.048). However, due to the limited number of studies, meta-regression was not conducted.

**Table 5:** Subgroup analysis and meta-regression of the association between processed meat consumption and the risk of development of ulcerative colitis.

Subgroup	*N*	Heterogeneity test		Estimate	
		*I^2^*	*p*	RR (95%CI)	*p*
Study design					
Cohort study	3	42.30%	0.177	1.292 (1.012, 1.649)	0.040
Case control study	4	99.60%	<0.001	2.193 (1.251, 3.842)	0.006
Dietary characteristics					
Western-style diet	3	42.30%	0.177	1.292 (1.012, 1.649)	0.040
Eastern-style diet	4	99.60%	<0.001	2.193 (1.251, 3.842)	0.006
Meat type					
Processed meat	5	96.10%	<0.001	1.292 (1.013, 1.649)	0.039
ham and sausage	1	/	/	0.800 (0.203, 3.150)	0.750
Grilled meat	1	/	/	14.079 (3.022, 65.596)	0.001
Dietary assessment tool					
T1	3	64.70%	0.059	1.352 (1.003, 1.821)	0.048
T2	2	90.40%	0.001	1.124 (0.775, 1.629)	0.538
T3	1	/	/	0.970 (0.916, 1.027)	0.295
T5	1	/	/	0.800 (0.203, 3.150)	0.750
Outcome measurement					
A	5	98.20%	<0.001	1.479 (1.051, 2.081)	0.025
B	2	0.00%	0.579	1.180 (0.842, 1.653)	0.337
Adjusted factors					
Geographical region					
Yes	3	54%	0.114	1.476 (1.049, 2.078)	0.026
No	4	95.00%	<0.001	1.182 (0.844, 1.656)	0.331
Family history of IBD					
Yes	1	/	/	14.079 (3.022, 65.596)	0.001
No	6	95.20%	<0.001	1.292 (1.013, 1.649)	0.039
Physical activity					
Yes	4	97.00%	<0.001	1.292 (1.012, 1.649)	0.040
No	3	73.40%	0.023	2.946 (1.373, 6.324)	0.006

N, number; IBD, inflammatory bowel disease; RR, inrelative risk; T1, country-specific validated food frequency questionnaires; T2, semi-quantitative food frequency questionnaires; T3, food frequency questionnaires; T5, self-administered questionnaire; A, self-reported questionnaire; B, individual interviews or self-reported questionnaire.

### 3.9 Publication bias and sensitivity analysis

Thirteen studies were included to examine the association between red meat intake and the risk of developing UC, prompting the construction of a funnel plot to assess publication bias (Supplementary Figure S3). Egger’s test revealed significant publication bias (*p* = 0.005). Therefore, a sensitivity analysis was conducted using the trim-and-fill method. After imputing five studies, the adjusted pooled effect size was RR = 1.026 (95% CI, 0.817–1.287), indicating no statistically significant association (*p* = 0.827). However, this estimate differed from the original pooled result, indicating potential instability in the findings.

### 3.10 Certainty of the evidence for main findings

According to the GRADE assessment, the overall certainty of the evidence was primarily rated as very low. This rating was mainly downgraded due to the complexity in categorizing meat types and substantial heterogeneity across studies (Supplementary Table S5).

## 4 Discussion

### 4.1 Summary of evidence

This systematic review included 18 cohort and case–control studies of moderate to high quality, encompassing a total of 1,384,024 participants. The findings suggest that, high intake of red meat may be significantly associated with an increased risk of developing UC. Conversely, the meta-analysis did not find a statistically significant association between processed meat consumption and UC risk. Regarding UC recurrence, the available evidence remains too limited to draw definitive conclusions; however, the current findings tentatively suggest that red and processed meat consumption may not be strongly associated with an elevated risk of disease recurrence. Furthermore, the overall certainty of evidence was rated as “very low” according to the GRADE framework, primarily due to substantial heterogeneity and the potential for misclassification bias. This high degree of heterogeneity, observed across most analyses, challenges the reliability of the point estimates and suggests that the true effect may differ.

With regard to red meat and UC risk, our findings confirmed a significant linear relationship. Specifically, an increase of 100 grams of red meat per day was associated with an approximately 65% increased risk of UC. Nevertheless, the presence of significant publication bias, as indicated by Egger’s test and the trim-and-fill adjustment, introduces uncertainty. The instability of the pooled estimates following this adjustment underscores that the observed association must be interpreted with considerable caution.

Recent animal studies suggest that high red meat intake may disrupt the colonic mucosal barrier in UC mouse models, increasing the expression of pro-inflammatory M1 macrophages and decreasing anti-inflammatory M2 macrophages, thereby disturbing the M1/M2 balance (20). Several biologically plausible mechanisms have been proposed to explain this link. Red meat is rich in compounds like carnitine and choline, which gut microbiota can metabolize into trimethylamine (TMA). Hepatic oxidation of TMA produces trimethylamine-N-oxide (TMAO), a metabolite implicated in promoting inflammatory processes that could contribute to UC pathogenesis (21). However, the biological mechanisms linking red meat to UC pathogenesis remain hypothetical and are primarily derived from animal models. It is essential to distinguish these mechanistic hypotheses from our evidence-based conclusions, as direct human evidence supporting the involvement of these pathways in UC development remains limited.

Subgroup analyses were conducted to explore the sources of heterogeneity for red meat. Heterogeneity was significantly reduced in analyses limited to studies that used country-specific validated food frequency questionnaires, suggesting that such tools may better capture regional dietary patterns and minimize measurement error. Geographic location also explained some heterogeneity, which may be attributed to differences in cooking practices. For example, the predominantly lower UC risk in regions like India, where meat is often minimally processed and cooked simply, contrasts with higher-risk Western countries (22). This implies that cooking methods may modify the risk associated with red meat consumption.

Contrary to common assumptions, our results suggest that processed meat intake may not increase UC risk to the same extent as red meat. This finding aligns with existing literature (23). This finding requires careful interpretation. A significant limitation in synthesizing the evidence on processed meat is the considerable variability in its definition across different studies and regions. The lack of a standardized definition likely introduces classification bias and complicates the interpretation of null results. For instance, more heavily processed meats—such as organ meats prepared with substantial additives and seasonings—may pose greater health risks than minimally processed products like simply seasoned dried meat (24). However, such gradations in processing levels are not consistently captured in current studies on processed meat. Furthermore, UC often has an acute onset with a relatively short preclinical phase, which may reduce the observable effects of processed meat consumption (25). Additionally, the substantial heterogeneity among the included studies and the potential for residual confounding factors—such as unadjusted total energy intake—preclude definitive conclusions that processed meat is not associated with UC risk.

Regarding UC recurrence, the analysis was based on a limited number of studies. The null findings are accompanied by wide confidence intervals and considerable heterogeneity, indicating that the evidence base is currently insufficient to draw definitive conclusions. Therefore, we refrain from making dietary recommendations for disease management based on these results, and more research is urgently needed in this area.

### 4.2 Strengths and limitations

This study investigates the association between red and processed meat consumption and the risk of UC, with a particular emphasis on the dose–response relationship. Nevertheless, several limitations should be acknowledged. First, this meta-analysis included both cohort and case–control studies; the retrospective nature of the latter may have introduced recall bias. Second, the majority of the included studies were conducted in Western populations, potentially limiting the generalizability of the findings to regions such as China and other Asian countries. Third, substantial heterogeneity among the included studies may compromise the robustness of the pooled estimates. To address this issue, subgroup analyses and meta-regression were performed to identify potential sources of heterogeneity.

### 4.3 Implications for future studies

Future prospective observational studies are warranted to investigate the association between UC incidence and the consumption of various types of processed meats, including smoked and cured products. Furthermore, due to individual dietary preferences and cultural habits, recommendations to reduce red and processed meat consumption in the general population have not been effectively adopted. Therefore, effective public health strategies are urgently needed to promote adherence to dietary recommendations. With regard to the risk of UC relapse, current evidence remains limited; thus, additional high-quality prospective cohort studies involving diverse populations and geographic regions are essential.

## 5 Conclusion

Current very-low-quality evidence suggests that red meat consumption may be associated with an increased risk of developing UC, following a linear dose–response pattern. However, there is currently insufficient evidence to support a causal link between red and processed meat intake and the risk of UC recurrence. Future research should prioritize well-designed prospective cohort studies to further elucidate the associations between red and processed meat consumption and both the incidence and recurrence of UC, as well as to investigate potential underlying mechanisms.

## Data Availability

The original contributions presented in the study are included in the article/Supplementary material, further inquiries can be directed to the corresponding authors.
