# Supplementary material for: The impact of red meat and processed meat consumption on the risk of development and relapse of ulcerative colitis: a systematic review and dose-response meta-analysis
Source: Front Nutr. 2025 Sep 12;12:1668302. doi: 10.3389/fnut.2025.1668302 (PMC12463588; doi:10.3389/fnut.2025.1668302)
Supplement: Supplementary file 1 [file Supplementary_file_1.docx]

Supplementary Material

The impact of red meat and processed meat consumption on the risk of development and relapse of ulcerative colitis: a systematic review and dose-response meta-analysis

Yangyang Zhang, Yitong Yu, Ziyun Jiang, Junhong Yu, Zeyang Zhang, Zhuojia An, Yanhong Du, Yiqing Mao, Lanshuo Hu^,^ , Xudong Tang , Yingpan Zhao, Tangyou Mao

目录

**[1 Supplementary Table S1:](#_Toc3357)** [Search strategy](#_Toc3357) **[2](#_Toc3357)**

**[2 Supplementary Table S2:](#_Toc9951)** [Exclusion literature list](#_Toc9951) **[3](#_Toc9951)**

**[3 Supplementary Table S3:](#_Toc25449)** [Results and adjusted factors of included studies for the risk of development of ulcerative colitis](#_Toc25449) **[6](#_Toc25449)**

**[4 Supplementary Table S4:](#_Toc15186)** [Results and adjusted factors of included studies for the risk of flare of ulcerative colitis](#_Toc15186) **[9](#_Toc15186)**

**[5 Supplementary Table S5:](#_Toc30146)** [Assess the overall quality of evidence using the Grading of Recommendations Assessments, Development and Evaluation](#_Toc30146) **[10](#_Toc30146)**

**[6 Supplementary Figure S1.](#_Toc25163)** [Forest plot of subgroup analysis of the association between red meat consumption and the risk of development of ulcerative colitis.](#_Toc25163) **[12](#_Toc25163)**

**[7 Supplementary Figure S2.](#_Toc29615)** [Forest plot of subgroup analysis of the association between processed meat consumption and the risk of development of ulcerative colitis.](#_Toc29615) **[13](#_Toc29615)**

**[8 Supplementary Figure S3.](#_Toc15210)** [Funnel plot of studies](#_Toc15210) **[14](#_Toc15210)**

# Supplementary Table S1: Search strategy

| **Databases** | **Search strategy** |
| --- | --- |
| CNKI | SU=红肉 + 发物 + 牛肉 + 羊肉 + 猪肉 + 加工肉 + 熏肉 + 腌肉 + 火腿 + 腊肉 + 香肠 + 培根 + 饮食 + 饮食模式 + 膳食 +食物 AND TKA="溃疡性结肠炎" |
| Wanfang | 主题:(红肉 OR 发物 OR 牛肉 OR 羊肉 OR猪肉 OR 加工肉 OR 熏肉 OR 腌肉 OR 火腿 OR 腊肉 OR 香肠 OR 培根 OR 饮食 OR 饮食模式 OR 膳食 OR 食物) AND 摘要: ("溃疡性结肠炎") |
| VIP | M=(红肉 OR 发物 OR 牛肉 OR 羊肉 OR猪肉 OR 加工肉 OR 熏肉 OR 腌肉 OR 火腿 OR 腊肉 OR 香肠 OR 培根 OR 饮食 OR 饮食模式 OR 膳食 OR 食物) AND U="溃疡性结肠炎" |
| Sinomed | ( "红肉"[常用字段:智能] OR "发物"[常用字段:智能] OR "牛肉"[常用字段:智能] OR "羊肉 OR猪肉"[常用字段:智能] OR "加工肉"[常用字段:智能] OR "熏肉"[常用字段:智能] OR "腌肉"[常用字段:智能] OR "火腿"[常用字段:智能] OR "腊肉"[常用字段:智能] OR "香肠"[常用字段:智能] OR "培根"[常用字段:智能] OR "饮食"[常用字段:智能] OR "饮食模式"[常用字段:智能] OR "膳食"[常用字段:智能] OR "食物"[常用字段:智能]) AND ""溃疡性结肠炎""[摘要:智能] |
| Yiigle Database | (主题=红肉 OR 发物 OR 牛肉 OR 羊肉 OR猪肉 OR 加工肉 OR 熏肉 OR 腌肉 OR 火腿 OR 腊肉 OR 香肠 OR 培根 OR 饮食 OR 饮食模式 OR 食物 OR 膳食*) AND 摘要=溃疡性结肠炎* |
| Pubmed | ((Colitis, Ulcerative[MeSH Major Topic]) OR (ulcerative colitis[Title/Abstract] OR Colitis Gravis[Title/Abstract])) AND ((meat[Title/Abstract] OR meat products[Title/Abstract] OR beef[Title/Abstract] OR lamb[Title/Abstract] OR mutton[Title/Abstract] OR pork[Title/Abstract] OR sausage[Title/Abstract] OR sausages[Title/Abstract] OR bacon[Title/Abstract]) OR (red meat [MeSH Major Topic])) |
| Embase | (meat:ti,ab,kw OR 'meat products':ti,ab,kw OR 'red meat':ti,ab,kw OR beef:ti,ab,kw OR lamb:ti,ab,kw OR mutton:ti,ab,kw OR pork:ti,ab,kw OR sausage:ti,ab,kw OR sausages:ti,ab,kw OR bacon:ti,ab,kw) AND ('ulcerative colitis':ti,ab,kw OR 'colitis gravis':ti,ab,kw) |
| The Cochrane Library | meat OR meat products OR red meat OR beef OR lamb OR mutton OR pork in Title Abstract Keyword AND ulcerative colitis OR Colitis Gravis in Title Abstract Keyword |
| Web of Science | (TS=(meat OR meat products OR red meat OR beef OR lamb OR mutton OR pork OR sausage OR sausages OR bacon)) AND AB=(ulcerative colitis OR Colitis Gravis) |
| International Clinical Trials Registry Platform | meat OR meat products OR red meat OR beef OR lamb OR mutton OR porkin Title Abstract Keyword AND ulcerative colitis OR Colitis Gravis in Title Abstract Keyword |

# Supplementary Table S2: Exclusion literature list

| **Study ID** | **Reason for exclusion** | **Title** |
| --- | --- | --- |
| BarreiroAM 2011 | conference papers | Influence Of Dietary Habits In Susceptibility For Inflammatory Bowel Disease: A Case Control Study |
| BikbavovaG 2019 | Non-red or processed meat exposure | The Impact Of Eating Habits On The Occurrence Of Ulcerative Colitis |
| CaiYJ 2020 | Non-red or processed meat exposure | 溃疡性结肠炎复发危险因素的研究 |
| ChenH 2022 | UC data inaccessible | Meat Consumption And All-Cause Mortality In 5763 Patients With Inflammatory Bowel Disease: A Retrospective Cohort Study |
| HeYH 2011 | Non-red or processed meat exposure | 溃疡性结肠炎患病及复发危险因素的病例—对照研究 |
| HeZY 2023 | Non-red or processed meat exposure | 溃疡性结肠炎的危险因素及中医体质相关性研究 |
| HigashiA 1991 | Japanese language | A case-control study of ulcerative colitis |
| HuiC 2021 | conference papers | Meat Consumption And All-Cause Mortality In 5763 Inflammatory Bowel Disease Patients: A Prospective Cohort Study |
| JantchouP 2010 | UC data inaccessible | Animal Protein Intake And Risk Of Inflammatory Bowel Disease: The E3N Prospective Study |
| JantchouP 2010b | duplicate records | Animal protein intake and risk of inflammatory bowel disease: the E3N Prospective Study |
| JowettSL 2002 | duplicate records | The Influence Of Dietary Factors On Relapse Of Ulcerative Colitis. An Observational Cohort Study |
| JowettSL 2005 | duplicate records | 饮食因素对溃疡性结肠炎临床病理过程的影响:一项前瞻性队列研究 |
| KhaliliH 2016 | conference papers | Dietary Iron And Red Meat Consumption, Autophagy, And Risk Of Crohn'S Disease And Ulcerative Colitis |
| KhaliliH 2020 | UC data inaccessible | Adherence to a Mediterranean diet is associated with a lower risk of later-onset Crohn’s disease: Results from two large prospective cohort studies |
| KonoS 1995 | conference papers | A Case-Control Study Of Ulcerative Colitis In Relation To Dietary And Other Factors In Japan |
| LeesCW 2023 | conference papers | Habitual Meat Intake Is Associated With Increased Risk Of Disease Flare In Ulcerative Colitis: Initial Results From The Predicct Study |
| LimketkaiBN 2022 | Non-red or processed meat exposure | Dietary Patterns And Their Association With Symptoms Activity In Inflammatory Bowel Diseases |
| MageeEA 2005 | type of research ineligible | Associations Between Diet And Disease Activity In Ulcerative Colitis Patients Using A Novel Method Of Data Analysis |
| NarulaN 2021 | duplicate records | Association Of Processed Food Intake With Risk Of Inflammatory Bowel Disease: Results From The Prospective Urban Rural Epidemiology (Pure) Study |
| OpsteltenJL 2019 | UC data inaccessible | Dietary Intake Of Patients With Inflammatory Bowel Disease: A Comparison With Individuals From A General Population And Associations With Relapse |
| OvergaardSH 2022 | UC data inaccessible | Impact Of Fibre And Red/Processed Meat Intake On Treatment Outcomes Among Patients With Chronic Inflammatory Diseases Initiating Biological Therapy: A Prospective Cohort Study |
| PetersV 2020 | UC data inaccessible | Dietary Intake Is Associated With Flare Development In Ibd Patients |
| PetersV 2022 | UC data inaccessible | Western And Carnivorous Dietary Patterns Are Associated With Greater Likelihood Of Ibd Development In A Large Prospective Population-Based Cohort |
| PieczyńskaJ 2019 | UC data inaccessible | Occurrence Of Dietary Risk Factors In Inflammatory Bowel Disease: Influence On The Nutritional Status Of Patients In Clinical Remission |
| PredaC 2020 | UC data inaccessible | Diet As An Environmental Trigger In Inflammatory Bowel Disease: A Retrospective Comparative Study In Two European Cohorts |
| PredaCM 2023 | UC data inaccessible | Impact Of Dietary Patterns In Inflammatory Bowel Disease Subtypes Versus Healthy Subjects: A Retrospective Cohort Study |
| RayG 2015 | UC data inaccessible | Association Of Dietary Factors With Ulcerative Colitis In India |
| RodriguezAD 2022 | type of research ineligible | A Diet High In Fruits And Vegetables During Biologic Induction May Improve Response To Biologics In Patients With Inflammatory Bowel Disease |
| SalgadoVCL 2011 | conference papers | Association Of Dietary Factors And Development Of Inflammatory Bowel Disease(Ibd) In Rio De Janeiro, Brazil |
| SayeghLN 2024 | UC data inaccessible | Nutritional Profile, Disease Severity, And Quality Of Life Of Patients With Inflammatory Bowel Disease: A Case-Control Study |
| SpehlmannME 2012 | UC data inaccessible | Risk Factors In German Twins With Inflammatory Bowel Disease: Results Of A Questionnaire-Based Survey |
| WangR 2022 | UC data inaccessible | 溃疡性结肠炎患者病情复发的相关因素研究 |
| WangYF 2013 | Non-red or processed meat exposure | Multicenter case-control study of the risk factors for ulcerative colitis in China |
| WengYJ 2019 | Non-red or processed meat exposure | 炎症性肠病患者肠道菌群失调分析及其与饮食关系的研究 |
| ZhangW 2012 | Non-red or processed meat exposure | 陕西地区人群溃疡性结肠炎发病危险因素的病例对照研究 |
| ZhongGXZZ 2008 | Non-red or processed meat exposure | 溃疡性结肠炎危险因素的病例对照研究 |
| ZhouMH 2012 | UC data inaccessible | 溃疡性结肠炎患病危险因素病例对照研究 |
| ZingerA 2023 | conference papers | High Red Meat Consumption Is Associated With Greater Risk Of Ulcerative Colitis Flare |

# Supplementary Table S3: Results and adjusted factors of included studies for the risk of development of ulcerative colitis

| **Study ID** | **Adjusted factors** | **Effect size** | **Reported estimate and 95%CI** |
| --- | --- | --- | --- |
| **Cohort study** | | | |
| Catherine 2022 | Centre,age at baseline,sex,smoking status,energy,alcohol,educational level,physical activity,BMI | HR | Total meat Q1 1  Q2 0.96(0.68-1.34) Q3 1.23(0.88-1.72) Q4 1.40(0.99-1.98) Red meat Q1 1 ; Q2 1.13(0.80-1.61) Q3 1.28(0.89-1.85) Q4 1.61(1.10-2.36) Processed meat Q1 1 ; Q2 1.10(0.81-1.49) Q3 0.97(0.71-1.34) Q4 1.18(0.84-1.65) |
| Emiy 2022 | Age,time-period,cohort,BMI,smoking status,physical activity,egular use of NSAIDs,history of appendectomy,family history of IBD ,daily servings of fruit,daily servings of vegetables,daily grams fiber,daily servings of red meat,and daily intake of n3:n6 PUFAs | HR | 0.99(0.92-1.07) |
| Neeraj 2021 | Age,sex,geographical region,education,alcohol intake,smoking status,physical activity,energy intake,BMI,waist to hip ratio,and urban versus rural location | HR | <1 serving/week:1 1 serving/week to <1 serving/day:1.77 (1.11 - 2.80)  ≥1 serving/day :2.19 (1.16 - 4.16) |
| Vanessa 2018 | Smoking,physical activity,obesity,and comorbidities as defined by 2 or more chronic conditions | OR | 1.007(0.871–1.166) |
| Hamed 2017 | Age,smoking,BMI at baseline,oral contraceptive use,menopausal hormone therapy,appendectomy,geographic latitude of residence at age 30,updated physical activity,cohorts,nonsteroidal anti-inflammatory drug’s use,updated fiber intake , and total caloric intake | HR | Red meat q1 1.00 q2 1.02(0.70-1.49) q3 1.11(0.75-1.63) q4 1.58(1.11-2.25) q5 1.10(0.72-1.65) Processed meat q1 1.00 q2 0.95(0.66-1.37) q3 0.98(0.70-1.38) q4 0.83(0.57-1.21) q5 1.12(0.77-1.62) |
| SongSY 2024 | Hygiene-related factors,self-rated health status,oral contraceptives,diseases history or medications at baseline related to antibiotics or NSAIDs | HR | Never, rarely or monthly:1 1-3 days per week:1.08 (0.73-1.59) ≥4 days per week:1.22 (0.78-1.90) |
| **Case-control study** | | | |
| Charles 2006 | Age, sex | OR | 2.62(1.37-5.03) |
| Farnaz 2022 | Age,sex,education,smoking,BMI,physical activity,status of marriage and total energy | RR | Red meat:0.99(0.98-1.01) Processed meats :0.97(0.91-1.02) |
| Kono 1993 | Sex,age at diease onset,study area,inpatient status | RR | Meat: Low:1;Moderate:2.0(1.0-3.8); High:1.3(0.6-3.0) Ham and sausage: Low:1; Moderate:1.1(0.6-1.9); High:0.8(0.2-3.1) |
| LiuXY 2021 | Occupational nature, level of job strain, living space area, breastfeeding, family history of IBD, gastrointestinal infections, autoimmune diseases, chronic diarrhea, dietary habits, fried foods, red meat, chicken, yogurt, fresh vegetables, fruits, grilled meat, coffee, honey, water intake, refrigerator use, alcohol consumption | OR | Red meat: 2.068(1.112-19.599) Grilled meat: 14.079(3.022-65.600) |
| Naomasa 2005 | Age,gender,study area,and other confounding covariates | OR | Q1:1.00 Q2:0.93(0.44-1.97) Q3:1.27(0.62-2.61) Q4:1.35(0.66-2.74) |
| Samaneh 2016 | Total energy intake,H.pylori infection,history of appendectomy,dietary fat,carbohydrate,and food groups intakes | OR | Processed meat T1:1 T2:1.13(0.41-2.51) T3:2.65(1.12-5.34) Red meat T1:1.00 T2:1.52(0.45-3.14) T3:2.52(1.40-6.24) |
| Giovanni 2010 | Age,sex,years of education,tobacco consumption, and BMI | OR | Low:1 Moderate:1.22 (0.45-3.32) High:0.63 (0.20-1.94) |

**Abbreviations:** IBD: inflammatory bowel disease, BMI: body mass index, NSAIDs: nonsteroidal anti-inflammatory drugs, PUFAs: polyunsaturated fatty acids, HR: hazard ratio, OR: odds ratio, RR: relative risk, CI: confidence interval, Q: quartiles of consumption, q: quintiles of consumption, T: tertiles of consumption.

# Supplementary Table S4: Results and adjusted factors of included studies for the risk of flare of ulcerative colitis

| **Study ID** | **Adjusted factors** | **Effect size** | **Reported estimate and 95%CI** |
| --- | --- | --- | --- |
| **Cohort study** | | | |
| Aaron 2013 | Age, sex, and prior surgery | OR | Red meat:0.88(0.68-1.14) Processed meat:0.97 (0.75-1.25) |
| Edward 2017 | NR | OR | T1 1  T2 Medium Intake：0.30 (0.13-0.74) T3 High Intake：0.94 (0.47-1.87) |
| Jowett 2004 | Age,sex,disease extent and duration,baseline SCCAI score,number of relapses in the year prior to recruitment,time since last relapse,average annual relapse frequency,medication use,including non-steroidal anti inflammatory drugs,smoking status, prior appendectomy | OR | Meat and meat products Low intake:1 Medium intake:1.37 (0.60-3.13) High intake:3.20 (1.31-7.79) Red and processed meat Low intake:1 Medium intake:2.16 (0.93-4.98) High intake:5.19 (2.09-12.9) |
| **Case-control study** | | | |
| LinM 2022 | The consumption of fish,seafood,vegetables,nuts,fruit | OR | 0.47(0.057-3.92) |
| Vera 2021 | Gender,age,BMI,all 25 food groups | OR | 1.005（1.001-1.009） |

**Abbreviations:** BMI: body mass index, SCCAI: short clinical colitis activity index, OR: odds ratio, T: tertiles of consumption, NR: not reported.

# Supplementary Table S5: Assess the overall quality of evidence using the Grading of Recommendations Assessments, Development and Evaluation

| The relationship between red or processed meat consumption and the risk of development of UC | | | | | | | | | | |
| --- | --- | --- | --- | --- | --- | --- | --- | --- | --- | --- |
| Patients or population: Healthy subjects | | | | | | | | | | |
| Exposure: Red or processed meat | | | | | | | | | | |
| Comparison: Control | | | | | | | | | | |
| **Outcomes** | **RR(95%CI)** | **N** | **Sample size** | **Risk of bias** | **Inconsistenoy** | **Indirectness** | **Imprecison** | **Publication bias** | **Upgrade quality of evidence** | **Quality of evidence** |
| red meat consumption and the risk of development of UC | 1.11(1.02,1.22) | 13 | 1,258,734 | No | serious^1^ | serious^2^ | No | Strongly suspected^3^ | upgrade^4^ | Very low |
| processed meat consumption and the risk of development of UC | 1.45(1.02,2.05) | 7 | 695,841 | No | serious^1^ | serious^2^ | No | - | upgrade^4^ | Very low |
| The relationship between red or processed meat consumption and the relapse risk of UC | | | | | | | | | | |
| Patients or population: Subjects with UC | | | | | | | | | | |
| Exposure: Red or processed meat | | | | | | | | | | |
| Comparison: Control | | | | | | | | | | |
| Outcomes | RR(95%CI) | N | Sample size | Risk of bias | Inconsistenoy | Indirectness | Imprecison | Publication bias | Upgrade quality of evidence | Quality of evidence |
| red meat consumption and the relapse risk of UC | 1.16(0.78,1.71) | 4 | 8841 | No | serious^1^ | serious^2^ | No | - | No | Very low |
| processed meat consumption and the relapse risk of UC | 1.54(0.65,3.63) | 3 | 7,363 | No | serious^1^ | serious^2^ | No | - | No | Very low |

**Abbreviations:** UC: ulcerative colitis, N: number, RR: relative risk.

1. The studies exhibited substantial heterogeneity (I² ＞ 50%).

2. The types of meat exposure were complex.

3. Significant publication bias was identified (P<0.05 in Egger test).

4. The study conducted a dose-response analysis.

# Supplementary Figure S1. Forest plot of subgroup analysis of the association between red meat consumption and the risk of development of ulcerative colitis.


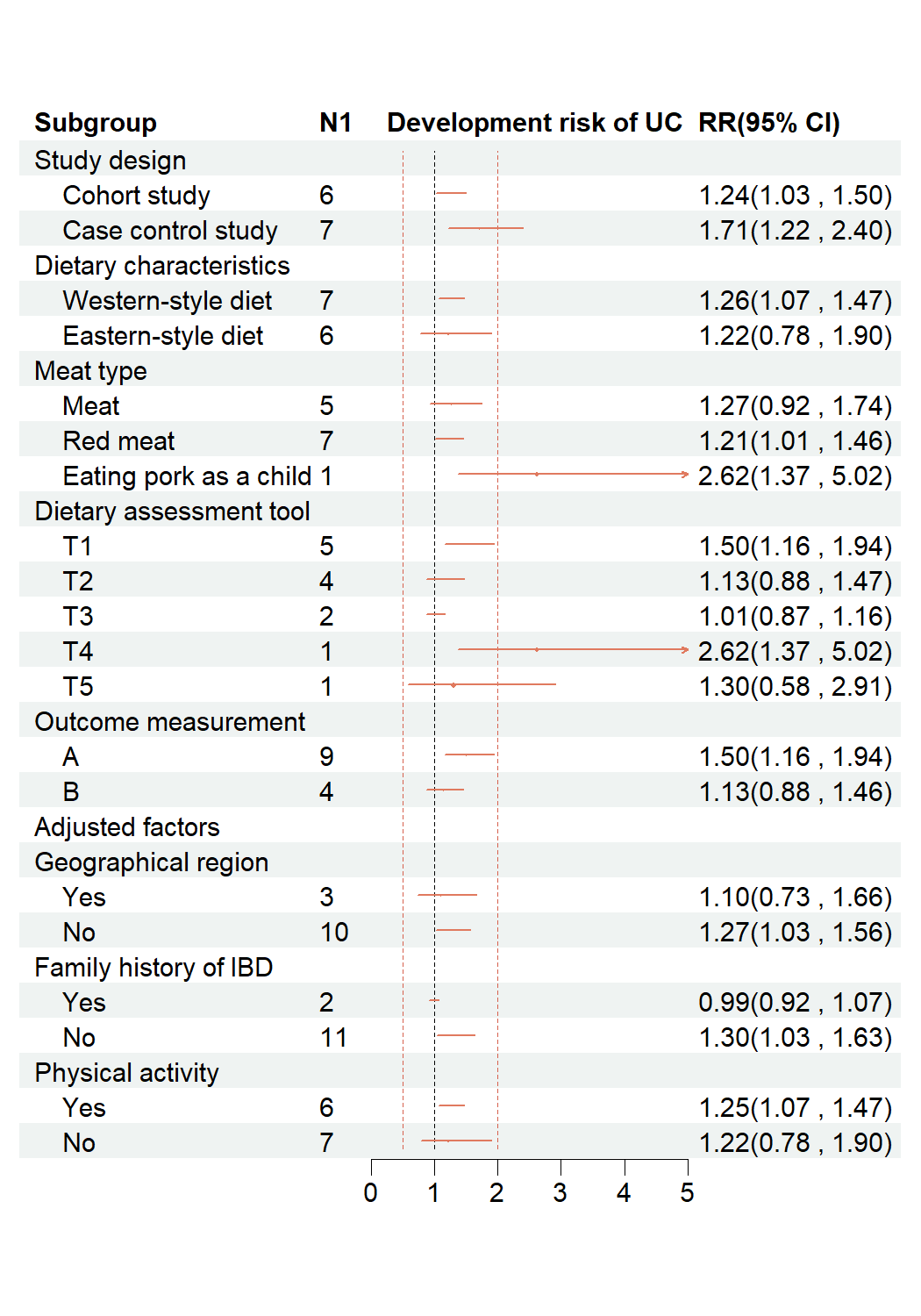


**Supplementary Figure S1. Forest plot of subgroup analysis of the association between red meat consumption and the risk of development of ulcerative colitis.** N: number; IBD: inflammatory bowel disease; RR: inrelative risk;T1: country-specific validated food frequency questionnaires, T2: semi-quantitative food frequency questionnaires, T3: food frequency questionnaires, T4: questionnaire about childhood dietary patterns, T5:self-administered questionnaire, A: self-reported questionnaire, B: individual interviews or self-reported questionnaire.

# Supplementary Figure S2. Forest plot of subgroup analysis of the association between processed meat consumption and the risk of development of ulcerative colitis.


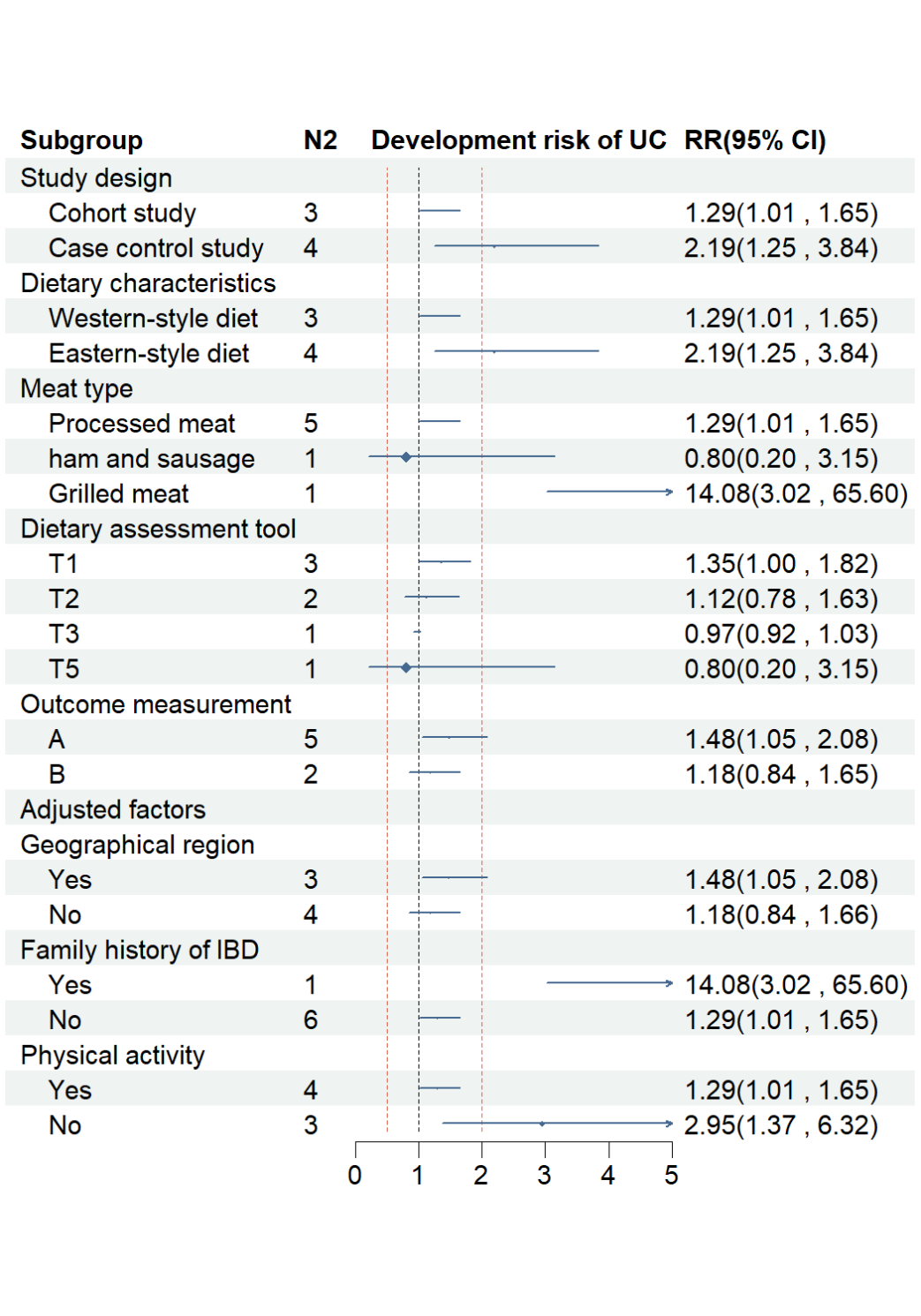


**Supplementary Figure S2. Forest plot of subgroup analysis of the association between processed meat consumption and the risk of development of ulcerative colitis.** N: number; IBD: inflammatory bowel disease; RR: inrelative risk;T1: country-specific validated food frequency questionnaires, T2: semi-quantitative food frequency questionnaires, T3: food frequency questionnaires, T4: questionnaire about childhood dietary patterns, T5:self-administered questionnaire, A: self-reported questionnaire, B: individual interviews or self-reported questionnaire.

# Supplementary Figure S3. Funnel plot of studies


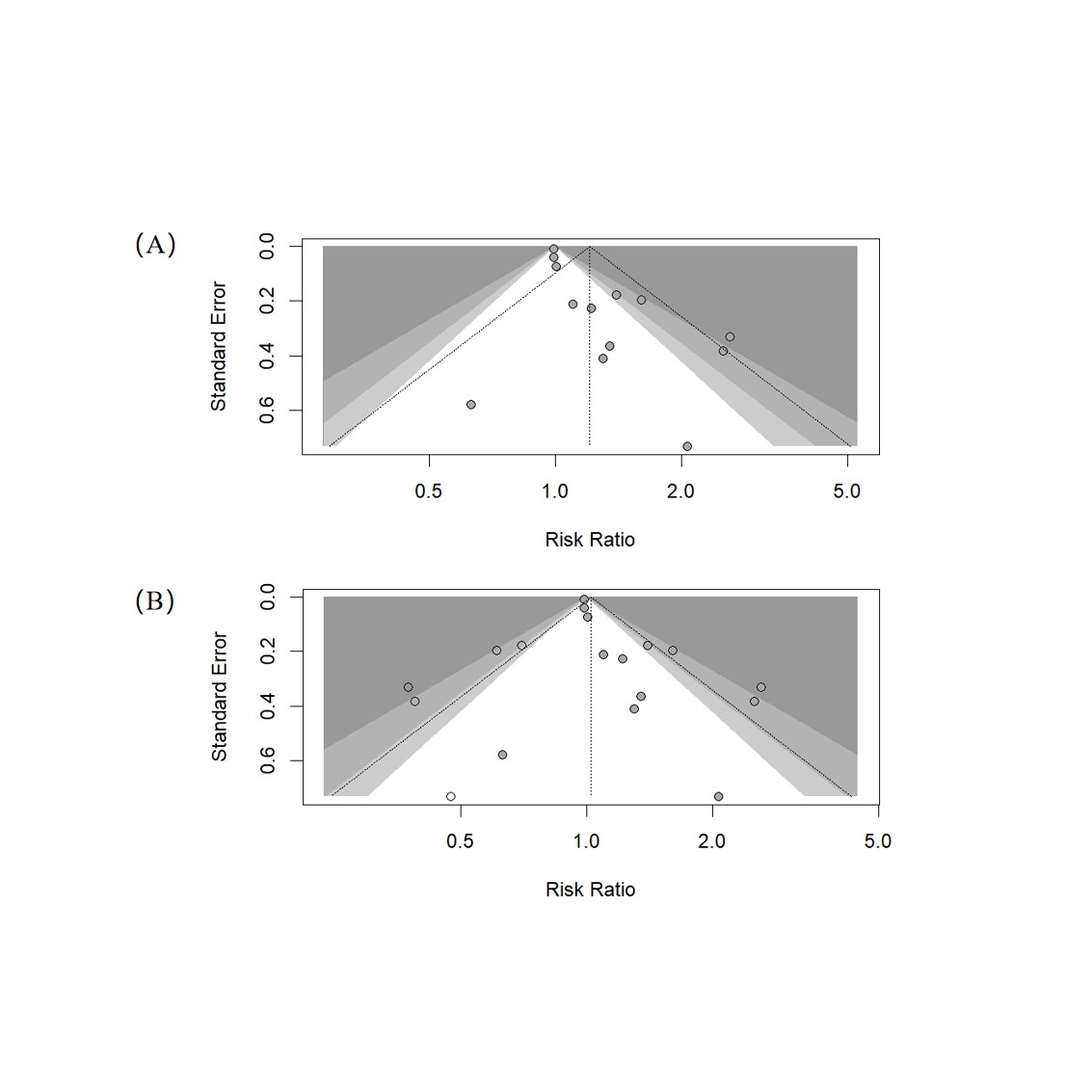


**Supplementary Figure S3. Funnel plot of studies.** (A)Funnel plot of studies on red meat intake and risk of UC; (B) Funnel plot of studies on red meat intake and risk of UC (trim-and-fill method).
